# The malnutrition in AECOPD and its association with unfavorable outcomes by comparing PNI, GNRI with the GLIM criteria: a retrospective cohort study

**DOI:** 10.3389/fnut.2024.1365462

**Published:** 2024-08-09

**Authors:** Xueyang Zhang, Yu Wang, Minmin Xu, Yuanyi Zhang, Quanjun Lyu

**Affiliations:** ^1^College of Public Health, Zhenghou University, Zhengzhou, Henan, China; ^2^Department of Respiratory and Critical Care Medicine, The First Affiliated Hospital of Zhengzhou University, Zhengzhou, Henan, China; ^3^Henan Key Laboratory for Pharmacology of Liver Diseases, Zhengzhou, China; ^4^Zhengzhou Shuqing Medical College, Zhengzhou, Henan, China

**Keywords:** acute exacerbation of chronic obstructive pulmonary disease, malnutrition, leadership initiative on malnutrition, geriatric nutritional risk index, prognostic nutritional index

## Abstract

**Introduction:**

The management of nutritional risk has garnered significant attention in individuals diagnosed with acute exacerbation of chronic obstructive pulmonary disease (AECOPD) due to the high prevalence of malnutrition and its correlation with unfavorable outcomes. While numerous rating scales exist to assist in assessment for both clinical and research purposes, there is considerable variability in the selection of scales based on the characteristics of the study participants and the study design. The objective of this study was to examine the efficacy of the Geriatric Nutritional Risk Index (GNRI) and Prognostic Nutritional Index (PNI) in identifying malnutrition and predicting prognosis in elderly AECOPD patients.

**Methods:**

From January 2022 to December 2022, a consecutive inclusion of elderly AECOPD patients admitted to the First Affiliated Hospital of Zhengzhou University was conducted. Diagnosing malnutrition in patients using PNI and GNRI, comparing the results with the diagnostic outcomes based on the Global Leadership Initiative on Malnutrition (GLIM) criteria through Receiver Operating Characteristic curves. Logistic regression analysis was employed to assess the risks associated with length of stay (LOS), hospitalization costs, and Charlson Comorbidity Index (CCI) based on GLIM, GNRI, or PNI.

**Results:**

A total of 839 elderly AECOPD patients were investigated in the study. The GNRI and PNI demonstrated a sensitivity of 89.5 and 74.1%, specificity of 77.2 and 66.4%, and an area under the curve of 0.834 and 0.702, respectively. The identification of high malnutrition-risk cases using the GLIM, GNRI and PNI were associated with a significant increase in the risk of LOS over 7 days [odds ratio (95% CI) for GLIM, GNRI, PNI: 1.376 (1.033–1.833); 1.405 (1.070–1.846); 1.875 (1.425–2.468)] and higher hospitalization expenses [OR (95% CI) for GLIM, GNRI: 1.498 (1.080–2.080); 1.510 (1.097–2.079)], but not with the CCI.

**Conclusion:**

According to our study, it is possible to use GNRI and PNI as alternatives to GLIM in the context of AECOPD, which makes it easier to identify malnutrition. The utilization of GNRI and PNI as alternatives to GLIM in the context of AECOPD enables the identification of malnutrition. The presence of malnourished individuals experiencing AECOPD is correlated with higher probabilities of extended hospital stays and escalated in-hospital expenses.

## 1 Introduction

An acute exacerbation is a frequent occurrence in individuals with chronic obstructive pulmonary disease (COPD), a gradually advancing respiratory condition marked by the decline of lung function and overall quality of life. These acute exacerbations of COPD (AECOPD) are prevalent and often result in hospitalizations and escalated healthcare expenses (1, 2). It is estimated that AECOPD contributes to 6% of global mortality rates and incurs annual costs exceeding $32 billion in the United States (3).

In recent years, there has been an increasing body of evidence suggesting that diet and nutrition may play a significant role in the development of COPD (4). Among patients with COPD, abnormal nutritional status and changes in body composition are prevalent comorbidities that have a detrimental effect on prognosis, including an increased risk of COPD exacerbation, depression, and mortality (5–7). Previous research has indicated that a substantial proportion of COPD patients, ranging from 30 to 60%, suffer from malnutrition (8, 9). Furthermore, elderly individuals are particularly susceptible to these complications as they age (10). Therefore, it is crucial to assess and address the nutritional status of COPD patients.

Despite the fact that malnutrition poses a major global health concern, the clinical diagnostic criteria have not been universally agreed upon (11). The Global Leadership Initiative on Malnutrition (GLIM) has developed a consensus-based report to establish universally applicable criteria for diagnosing malnutrition (12). Furthermore, various quantitative nutritional assessment tools have been employed to screen and evaluate malnutrition. The Geriatric Nutritional Risk Index (GNRI) is a validated nutrition-related index that predicts clinical outcomes in elderly patients (13). Similarly, the Prognostic Nutrition Index (PNI) is utilized to evaluate the nutritional status of surgical patients, anticipate surgical risks, and make prognostic assessments (11). However, the validation of quantitative nutritional tools in relation to the standard malnutrition diagnosis criteria as a reference for patients with AECOPD and its impact on in hospital outcomes remains unexplored.

In this study, the GNRI and PNI were examined for their effectiveness in detecting GLIM-defined malnutrition and predicting prognosis in elderly AECOPD patients.

## 2 Methods

This research constitutes a single-center observational cohort study, focusing on elderly patients admitted to the First Affiliated Hospital of Zhengzhou University due to acute exacerbation of chronic obstructive pulmonary disease between January 2022 and December 2022. The study received ethical approval from the Ethics Committee of the First Affiliated Hospital of Zhengzhou University.

### 2.1 Study population

The inclusion criteria for this study were as follows: (1) individuals aged 60 years or older; and (2) individuals diagnosed with AECOPD based on the guidelines provided by the Chinese Expert Consensus (14): symptoms such as dyspnea, chronic cough or sputum production, a history of lower respiratory tract infections with recurrent episodes can be considered as indicative of COPD; further deterioration of respiratory function may lead to the diagnosis of AECOPD. Conversely, the exclusion criteria encompassed the following: (1) individuals with advanced malignant tumors or end-stage diseases in organs other than the lung; (2) individuals with communication disorders, such as severe hearing impairment or cognitive impairment; (3) individuals participating in other research projects related to nutrition; and (4) individuals lacking any of the necessary data for analysis.

### 2.2 Data collection

Clinical, demographic, and laboratory data were acquired through the process of inquiry and retrieval from electronic medical records. Upon admission, pertinent information such as sex, age, body weight, height, and nutritional status was gathered. Throughout the hospital stay, clinical data encompassing diagnosis, comorbidity, laboratory tests and length of stay, hospitalization costs, were extracted from the electronic medical records system. Additionally, nutrition-related laboratory tests, namely hemoglobin (HB), serum albumin (ALB), and lymphocyte-monocyte ratio (LMR), were included in the data collection process.

### 2.3 Nutritional assessment

Three screening tools (GLIM, PNI and GNRI) were used to assess elderly AECOPD patients’ nutritional status.

The GLIM model, as described by previous study (12), consists of a two-step process for the diagnosis of malnutrition. The initial step involves conducting malnutrition risk screening, utilizing the Nutritional Risk Screening 2002 (NRS 2002) in this particular study. A NRS 2002 score of ≥3 indicates a risk of malnutrition. The second step necessitates the presence of at least one of the three phenotypic criteria (non-volitional weight loss, low BMI, and reduced muscle mass) and one of the two etiologic criteria (reduced food intake or assimilation, and disease burden/inflammation) for the confirmation of malnutrition diagnosis.

The PNI and GNRI are objective screening tools utilized to evaluate the nutritional status and forecast patient prognosis (15). The former is determined by the levels of serum albumin and lymphocytes (16), with the PNI calculated as albumin (g/L) + 5 × lymphocyte count (×109/L). In accordance with previous research, a threshold value of 45 was employed in this study, designating scores ≥45 as indicative of “well-nourished” individuals, while scores <45 were classified as “malnourished”. The formula for GNRI was as follows (13): GNRI = (1.489 × albumin (g/l) + 41.7 × weight (kg)/ideal body weight (kg)). The Lorentz equation was employed to determine the ideal body weight, with separate calculations for women and men. If the ratio of weight to ideal body weight exceeded or equaled 1.0, it was adjusted to 1. For the purposes of this study, the classification system was simplified to two categories: no risk (GNRI >98) and at risk (GNRI ≤98).

### 2.4 Statistical analysis

IBM SPSS Statistics 24 was utilized for conducting statistical analyses, with a predetermined level of significance set at 0.05. Continuous variables were expressed as means ± standard deviation (SD), while categorical variables were presented as frequencies and percentages. To compare the baseline characteristics of the two groups, namely those with malnutrition and those without malnutrition, a Student’s *t*-test was employed for continuous variables, whereas the Chi-Squared test was utilized for categorical variables. Receiver operating characteristic (ROC) curves were employed to assess the diagnostic efficacy of GNRI and PNI in relation to GLIM-defined malnutrition. Logistic regression analysis was conducted to assess the associations between malnutrition, as determined by the aforementioned tools, and the risks of extended length of stay (LOS), increased hospitalization costs, and Charlson Comorbidity Index (CCI).

## 3 Results

A total of 839 patients were included in our study. The characteristics of these patients are presented in Table 1. Among the patients diagnosed with AECOPD, the average age was determined to be 72.06 ± 7.54 years, with males comprising 80.5% of the entire participant pool. Additionally, the LOS was found to be 8.96 ± 6.42 days and the hosptialization costs per day was ¥2347.22 ± 5535.20. Furthermore, it was observed that 286 patients (34.1%) met the criteria for malnutrition as defined by the GLIM. The prevalence rates of malnutrition, as determined by the GNRI and the PNI, were found to be 47.4 and 24.7%, respectively.

**Table 1:** Demographic and clinic characteristics of participants.

Variables	Values (*n* = 839)
Age, year	72.06 (7.54)
Male, *n* (%)	675 (80.50)
Height, cm	167.27 (6.66)
Weight, kg	62.36 (10.47)
Body mass index, *n* (%)	
<18.5 kg/m^2^	134 (16)
18.5–24.9 kg/m^2^	540 (54.5)
25.0–29.9 kg/m^2^	141 (16.8)
>30.0 kg/m^2^	22 (2.6)
ALB, g/L	34.98 (4.78)
HB, g/L	131.06 (22.19)
LMR	1.64 (2.69)
LOS, days	8.96 (6.42)
hospitalization costs per day, ¥	2347.22 ± 5535.20
CCI, *n* (%)	
4	145 (17.2)
5	307 (36.6)
6	204 (24.3)
7	148 (17.6)
8	35 (4.2)
GLIM-defined malnutrition, *n* (%)	286 (34.1)
PNI <45, *n* (%)	398 (47.4)
GNRI ≤98, *n* (%)	207 (24.7)

ALB, albumin; Hb, hemoglobin; LMR, lymphocyte-to-monocyte ratio; LOS, length of stay; CCI, Charlson comorbidity index; GLIM, global leadership initiative on malnutrition; PNI, prognostic nutritional index; GNRI, geriatric nutritional risk index.

Table 2 presents the observed variations in characteristics and classical nutritional markers between two distinct groups, which were divided based on three nutritional screening tools. In comparison to the normal group, the malnutritional group identified by GLIM, PNI, and GNRI exhibited significantly higher age and lower body weight, body mass index, albumin, hemoglobin, lymphocyte-to-monocyte ratio and length of stay (*p* < 0.05). Furthermore, malnourishment was found to be more prevalent among male patients when identified by PNI or GNRI, but not when identified by GLIM. Additionally, malnourishment identified by GNRI, but not PNI or GLIM, was associated with higher hospitalization costs per day.

**Table 2:** Comparison of the basic profile of AECOPD patients in the malnourished and normal groups diagnosed by the three tools.

variables	PNI			GNRI			GLIM		
	Malnutrition (*n* = 399)	Normal (*n* = 440)	*p*	Malnutrition (*n* = 382)	Normal (*n* = 457)	*p*	Malnutrition (*n* = 286)	Normal (*n* = 553)	*p*
Age, years	73.42 (7.49)	70.84 (7.37)	<0.001	73.11 (7.32)	71.19 (7.61)	<0.001	74.80 (7.72)	70.64 (7.04)	<0.001
Male, *n* (%)	333 (83.67)	342 (77.73)	0.03	325 (85.08)	350 (71.87)	0.002	240 (83.92)	435 (78.66)	0.076
Weight, kg	60.31 (10.34)	64.22 (10.25)	<0.001	55.91 (8.16)	67.76 (9.04)	<0.001	54.95 (9.62)	66.20 (8.69)	<0.001
BMI, kg/m^2^	21.43 (3.46)	23.05 (3.36)	<0.001	19.87 (2.53)	24.29 (2.88)	<0.001	19.54 (3.16)	23.69 (2.76)	<0.001
ALB, g/L	34.66 (3.82)	40.98 (3.37)	<0.001	34.64 (3.94)	40.78 (3.45)	<0.001	34.68 (4.68)	39.69 (3.85)	<0.001
HB, g/L	125.98 (24.33)	135.84 (18.79)	<0.001	126.37 (24.42)	135.11 (19.19)	<0.001	125.80 (24.90)	133.83 (20.11)	<0.001
LMR	0.66 (0.59)	0.34 (0.18)	<0.001	0.61 (0.56)	0.40 (0.31)	<0.001	0.63 (0.62)	0.42 (0.32)	<0.001
LOS, days	9.85 (6.51)	8.15 (6.24)	<0.001	9.52 (6.41)	8.49 (6.40)	0.021	9.77 (6.06)	8.54 (6.57)	0.009
Hospitalization costs	2572.03 (379.30)	2144.84 (124.09)	0.098	2782.89 (408.93)	1984.00 (79.02)	0.004	2588.92 (448.20)	2222.66 (174.11)	0.101
CCI	5.58 (1.13)	5.52 (1.07)	0.423	5.57 (1.10)	5.52 (1.10)	0.550	5.56 (1.12)	5.54 (1.09)	0.747

BMI, body mass index; ALB, albumin; Hb, hemoglobin; LMR, lymphocyte-to-monocyte ratio; LOS, length of stay; CCI, Charlson comorbidity index; GLIM, global leadership initiative on malnutrition; PNI, prognostic nutritional index; GNRI, geriatric nutritional risk index.

Table 3 presents the sensitivity, specificity, positive predictive value, negative predictive value, positive likelihood ratio, negative likelihood ratio, and area under the curve (AUC) of PNI and GNRI in the identification of malnutrition as defined by GLIM. Both PNI and GNRI demonstrated acceptable diagnostic performance for malnutrition, as evidenced by their respective sensitivity values of 0.741 and 0.895, specificity values of 0.664 and 0.772, and AUC values of 0.702 and 0.834.

**Table 3:** Cross tabulation of the results of PNI, GNRI, and GLIM diagnostic criteria for the diagnosis of malnutrition.

	PNI	GNRI
Sensitivity	0.741	0.895
Specificity	0.664	0.772
Positive predictive value	0.664	0.772
Negative predictive value	0.259	0.105
Positive likelihood ratio	2.205	3.925
Negative likelihood ratio	0.39	0.136
K value	0.370	0.617
AUC	0.702	0.834

PNI, prognostic nutritional index; GNRI, geriatric nutritional risk index; AUC, Area Under Curve.

**Figure 1:**
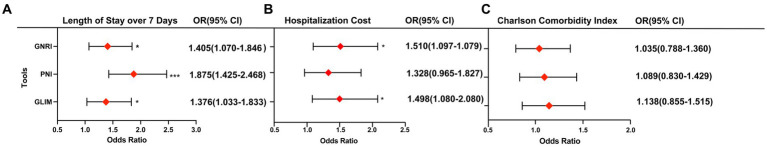
Association of malnutrition diagnosed by three tools with length of stay over 7-day **(A)**, hospitalization costs **(B)** and CCI **(C)**. Odds ratio are adjusted for age, sex, and BMI. *p < 0.05; ***p < 0.001.

The multivariate analysis revealed that malnutrition, as indicated by GNRI (OR = 1.405, 95% CI 1.070–1.846, *p* = 0.015), PNI (OR = 1.875, 95% CI 1.425–2.468, *p* < 0.001), and GLIM (OR = 1.376, 95% CI 1.033–1.833, *p* = 0.029), was significantly associated with an increased risk of exceeding 7-day LOS (Figure 1). Age, sex, and BMI were adjusted as covariates in the multivariable analysis. Collinearity regression results indicated that there was no collinearity between age, sex, and BMI, allowing them to be used as covariates in the analysis. Furthermore, malnutrition identified by GNRI (OR = 1.510, 95% CI 1.097–2.079, *p* = 0.011) and GLIM (OR = 1.498, 95% CI 1.080–2.080, *p* = 0.016) was found to be associated with a higher risk of hospitalization costs. However, the malnutrition identified by these three assessment tools did not show any significant association with the CCI.

## 4 Discussion

Patients were assessed using three different tools (GLIM, PNI, and GNRI) to diagnose malnutrition in our study. The results indicated that the rates of nutritional risk in elderly hospitalized populations with AECOPD, as determined by GLIM, PNI, and GNRI, were 28.5, 47.4, and 45.5%, respectively. Previous studies have reported malnutrition rates of 30–60% in COPD patients (8, 9, 17). AECOPD, defined as a sudden exacerbation of respiratory symptoms requiring additional treatment (18, 19), may contribute to a higher nutritional risk compared to patients with typical COPD. In conclusion, our findings are generally consistent with these previous studies.

Malnutrition is characterized as a state of inadequate nutrition that detrimentally impacts the structural or functional capabilities of the body, encompassing a decrease in caloric and protein intake (20). In the context of patients with acute exacerbations of chronic obstructive pulmonary disease (AECOPD), the heightened energy expenditure resulting from respiratory effort can contribute to malnutrition. Moreover, humoral factors, including inflammatory cytokines, adipokines, and hormones, may also serve as potential etiological factors for malnutrition in AECOPD patients (21, 22). Additionally, malnutrition may be linked to reduced overall physical activity or diminished appetite stemming from depressive tendencies in individuals with AECOPD (23). The presence of malnutrition has the potential to compromise the patient’s immune defenses and expedite the progression of diseases. Additionally, it can result in a decline in skeletal muscle mass and functionality, a decrease in diaphragm mass and thickness, ultimately culminating in respiratory failure (20). Consequently, it is imperative to promptly address nutritional status and prevent unfavorable prognoses associated with malnutrition.

Despite the widespread acceptance of the GLIM diagnostic criteria for malnutrition as published in 2018, the clinical implementation is deemed inadequate due to the complexity of the steps involved and the lack of clarity in defining factors such as inflammation or disease burden (24).

Furthermore, the prevalence of malnutrition in AECOPD is notably high, yet there has been limited research conducted on nutritional screening tools specifically for this population. This study aimed to examine the efficacy of two quantitative nutritional tools, namely the GNRI and the PNI, in the detection of malnutrition as defined by the GLIM in elderly patients with AECOPD. The GNRI exhibited higher sensitivity (89.5%), satisfactory specificity (77.2%), and a larger AUC value of 0.834. Similarly, the PNI demonstrated good sensitivity (74.1%), specificity (66.4%), and an AUC of 0.702. These findings suggest that either the PNI or GNRI may serve as a viable alternative tool for assessing malnutrition in this patient population. The agreement (k-value = 0.617) between the GNRI and GLIM can be attributed to the inclusion of serum albumin, current weight, and ideal weight in the GNRI. Serum albumin levels have long been considered indicative of nutritional status and systemic inflammation in patients with AECOPD. Consequently, serum albumin serves as a supportive proxy measure of inflammation and is one of the etiologic criteria of GLIM (25, 26). Additionally, the body weight/ideal body weight ratio provides a macroscopic description of skeletal muscle mass (13). In contrast, the PNI solely incorporates two laboratory indicators (serum albumin and lymphocytes), without considering any anthropometric measurements.

In the current investigation, the utilization of GLIM, PNI, and GNRI as diagnostic tools for identifying malnutrition were found to be associated with increased length of stay exceeding 7 days and higher hospitalization costs. However, these diagnostic measures were not found to be predictive of the results of CCI. Several potential explanations may account for these findings. Prior research conducted on hospitalized patients has revealed a significant prevalence of malnutrition upon admission, which has been shown to have a detrimental effect on LOS and contribute to escalated hospitalization costs (27). The analysis of cost estimation data from various countries indicates that the direct cost of treating AECOPD is a substantial portion of the healthcare budget (28–30). Advanced AECOPD commonly presents with multiple comorbidities, leading to a greater symptom burden and poorer outcomes (31). Additionally, malnutrition has been linked to longer hospital stays and higher costs (27), and it also contributes to the progression of AECOPD. However, it is important to note that our findings only suggest a trend rather than statistical significance in the association between malnutrition and an increase in the CCI, potentially due to the limited sample size.

## 5 Conclusion

Malnutrition can be detected using GNRI and PNI, which are alternatives to GLIM, in the context of AECOPD. An elevated probability of prolonged hospital stays and escalating hospitalization costs is associated with the malnourished AECOPD. Further research is needed to verify our conclusion.

## Data availability statement

The original contributions presented in the study are included in the article/supplementary material, further inquiries can be directed to the corresponding author.

## Ethics statement

The studies involving humans were approved by the Scientific Research and Clinical Trial Ethics Committee of the First Affiliated Hospital of Zhengzhou University. The studies were conducted in accordance with the local legislation and institutional requirements. The human samples used in this study were acquired from a by- product of routine care or industry. Written informed consent for participation was not required from the participants or the participants' legal guardians/next of kin in accordance with the national legislation and institutional requirements.

## Author contributions

XZ: Data curation, Formal analysis, Methodology, Writing – original draft, Writing – review & editing. YW: Data curation, Methodology, Writing – original draft. MX: Data curation, Resources, Writing – original draft. YZ: Data curation, Resources, Writing – original draft. QL: Supervision, Writing – review & editing.
